# First aid knowledge, attitude, practice, and associated factors among kindergarten teachers of Lideta sub-city Addis Ababa, Ethiopia

**DOI:** 10.1371/journal.pone.0194263

**Published:** 2018-03-13

**Authors:** Gemechu Ganfure, Gemechu Ameya, Ababe Tamirat, Bikila Lencha, Dereje Bikila

**Affiliations:** 1 Department of Midwifery, Goba Referral Hospital Madda Walabu University, Goba, Ethiopia; 2 Department of Medical Laboratory Science, College of medicine and health sciences, Arba Minch University, Arba Minch, Ethiopia; 3 Department of Nursing, College of medicine and health sciences, Hawassa University, Hawassa, Ethiopia; 4 Department of public health, Goba Referral Hospital Madda Walabu University, Goba, Ethiopia; 5 Department of Nursing, College of medicine and health sciences, Arsi University, Asella, Ethiopia; University of Campania, ITALY

## Abstract

**Background:**

Injuries are very common and can occur at any point of time in a day. Unintended injuries in kindergarten children are the most common and need immediate life saving care which is known as first aid. This study aimed to investigate knowledge, attitude, practice, and associated factors of first aid among kindergarten teachers of Lideta sub-city Addis Ababa, Ethiopia.

**Method:**

A cross-sectional study was conducted among kindergarten teachers. Data was collected using pretested, structured and self-administered questionnaire S1 File. The collected data was entered in to Epi Data version 3.1 software and analyzed using SPSS version 20. Logistic regression analysis was used to identify association between kindergarten teachers’ knowledge and attitudes towards first aid and different variables. Odds ratios with 95% CI and p<0.05 were computed to determine the presence of the association.

**Result:**

One hundred and ninety-four teachers participated in the study with a response rate of 95%. Only 40% of the teachers were knowledgeable and 75% of them had positive attitude for first aid. Eighty percent of teachers encountered with children in need of first aid. Kindergarten teachers older than 35 years [AOR = 4.2, 95%CI: (1.02, 16.9)], five years’ experience [AOR = 3.1, 95%CI: (1.2, 7.6)], having previous first aid training [AOR = 3.1, 95%CI: (1.2, 7.7)], source of first aid information and teachers serving in private kindergarten are associated with having knowledge of first aid. Long time experience, type of kindergarten, previous training, and exposure to children in need of first aid were positive association with attitude towards first aid.

**Conclusion:**

Low first aid knowledge and high positive attitude among kindergarten teachers. Having long time experience, being older age, previous first aid training, and serving in private kindergarten were positively associated with first aid knowledge and positive attitude. Creating awareness and including first aid courses in the kindergarten teachers’ curriculum need to be considered.

## Introduction

Accidents can happen anywhere at any time. The consequence of unintentional accident can be life threatening. Unintentional accident needs immediate and appropriate life saving care before the affected person get major treatment [1]. This life saving care or first aid is an assessment and interventions that can be carried out by a person nearby immediately with minimal or without medical equipment [2]. Therefore, this makes it important to have basic knowledge of first aid. The ultimate goal of first aid is to stop or to reverse the possible harm at a given time before reaching the appropriate health care center [3]. First aid knowledge is methods and techniques that used perform practices related to prevention and immediate response to health emergencies. It can be given in all areas such as household, schools, workplace, and recreational areas. Beyond health matters, first aid knowledge also increases the social responsibility of the society and strengthens values [4].

Study showed that two-thirds of the children had experienced at least one unexpected injury in a year. Majority of injuries occurred in child whose parents did not believe that it is possible to prevent unintentional injuries [5]. Qualitative study showed that child unintentional injuries at home may results from perception that some injuries were an expected part of child growth. Facilitators for injury may be mitigated by teaching children about injury risks and supervision [6]. The other study showed that young children from low income families are more affected by unintentional injury [7]. Injury prevention message should be basic and concise, and should be in written and pictorial form. Social networks are important in raising awareness and adherence to child safety advice [8]. In Ethiopia, there is limited information about child injury. The national information about injuries and accidents showed that about three percent of households reported having at least one member who was injured or killed in one year period before the survey. Urban residents are more likely facing injury than rural residents. Injure of children less than nine years old accounts one third of the total injure recorded in the household members [9].

Study showed that majority of school related injuries occurred in school playground, during physical activities, on school building, while going or coming back from the school [10]. Children have growing and developing bodies that are susceptible to accidental injuries. They spend a significant portion of their day in kindergarten. Therefore, paediatric emergencies due to unintentional accident that may results physical injuries are more likely to occur in this setting. In the absence of parents or caregivers, kindergarten is the best place to give care to children [11, 12]. For this reason, while a school is being established, there must be a person who is trained on first aid, and should be present in regular bases [13, 14]. In kindergartens, the teacher has crucial role in caring for children, supervising and preventing them from potential of health hazards. The kindergartner should be well trained on first aid and emergency control to save children’s lives. Furthermore, kindergartners should have adequate knowledge and skills about what they are doing [15, 16, 17].

Majority of public school teachers were deficient in both training and knowledge of emergency care [18]. Most incidental injury occurred in school demand first aid care. Despite the accidents that are common in school children, previous studies show that the knowledge, attitude and practice of first aid are low [2, 5, 19]. Studies conducted on knowledge, attitude and practice (KAP) of first aid in kindergarten teachers are very limited. Therefore, since this study is focused on knowledge, attitude and practice and associated factors of kindergartens teachers for first aid, the finding would used as important information for different concerned bodies. For ministry of education, this study can be used as input to plan appropriate interventions and modification of kindergartner teacher training curriculum. This study also helps to identify kindergartners KAP status that show area of improvement. Policy maker may use to generate a new policy concerning child care in kindergartens. Finally, this study is used for promotion of appropriate life saving treatment at Kindergartens. The aim of this study is evaluate knowledge, attitude and practice of first aid and its associated factors among kindergarten teachers of Lideta sub-city, Addis Ababa.

## Methods

### Ethics statement

Ethical clearance and approval was obtained from Nursing and Midwifery department ethical review committee of Addis Ababa University. Official letter was written to Addis Ababa city education bureau, and permission was obtained from Lideta sub-city education bureau. Both oral and written informed consents were obtained from respondents who participated in the study. In addition, all the responses were kept confidential and anonymous.

### Study design and settings

Institution based cross-sectional study design was employed. The study was conducted in Lideta sub-city Addis Ababa, Ethiopia. Among thirty-eight kindergartens found in the sub-city, 23 of them selected randomly and proportionally allocated from public, private, and community schools. Participants were selected randomly from proportionally allocated kindergartens. All randomly selected teachers working in the selected kindergartens and had willingness to participate in the study were included in the study.

### Data collection tools and procedures

The sample size was calculated using single population proportion formula: n = (Zα/2)^2^ * p(1-p) /d^2^, by considering the following assumptions: Zα/_2_ = 1.96 (Standard score value for 95% confidence level), p = 0.5 (Prevalence of KAP of first aid 50%, since there is no similar study conducted in the study setting), and d (tolerated margin of error) = 5%. Then finite population correction formula was applied for the study population is less than 10,000. Finally 10% non-response rate was added and the total number of study participants became 204.

Data was collected by using pretested, structured and self-administered questionnaire. The questionnaire contains four parts. The first part of question contain questions which used to assess socio-demographic characteristics of kindergarten teachers such as sex, age, level of education, year of experience, marital status, type of kindergarten they teach, and previous first aid training. The second part of the questionnaire has twelve questions that used to assess kindergartner first aid knowledge. The third part has questions and used to assess kindergarten teachers’ attitude towards first aid. Then the last one contains seventy questions which used to assess practice of kindergarten teachers on first aid. The knowledge questions were adopted and modified from American academy of pediatrics [20]. Attitude and practice questions of kindergarten teachers on first aid were developed after pertinent literatures review [2, 5, 6, 16, 18, 19, 21]. In addition to this, the content validity of the questionnaire was checked by using content validity index method [22]. Data quality was ensured during collection, coding, entry and analysis. The English version questionnaire was translated into the local language (Amharic) and then Amharic version was back translated to English version to ensure consistency. Before actual data collection, pretest was done on 5% of similar population out of study area in Kirkos Sub-city Addis Ababa to assess the suitability of the content, readability, feasibility, clarity, sequence and flow of the questionnaire. The filled questionnaires were checked for completeness by data collectors, supervisors and principal investigator on a daily basis.

### Statistical analysis

Data was first checked manually for completeness and then coded and entered into Epi-Data version 3.1 statistical software and cleaned thoroughly before transported to SPSS version 20 for further analysis. Knowledge about first aid was measured by using twelve knowledge questions and dichotomize to knowledgeable and non knowledgeable. A score of mean value and above to knowledge related questions was considered as knowledgeable, while less than the mean value indicated as non knowledgeable. In knowledge model independent variables were age of participants, educational level, year of experience, type of kindergartner they teach, marital status, previous first aid training, attitude towards to first aid, and source of first aid information. The variables were selected based on previous literature.

Kindergarten teachers’ attitude towards first aid was assessed by using four point Likert scale as individuals responding strongly agree and agree for positive attitude were given scores of 4 and 3, respectively, and score of 2 and 1 were given for those who responded as disagree and strongly disagree respectively and the order of scoring for negative statements was reversed. Then, the score was dichotomized into positive and negative attitude for each question and for the overall attitude status ninety percent of questions and above was considered as a cutoff point. In attitude model independent variables were knowledge, age, marital status, educational level, year of experience, type of kindergartner they teach, previous first aid training, previous exposure to children in need of first aid, and source of first aid information. Descriptive statistics were used to summarize practice of kindergarten teachers for first aid required cases.

Logistic regression analysis was used to identify factors associated with the attitude and knowledge of kindergarten teachers for first aid. Univariate logistic regression analysis was used to identify potential associated factors between dependent and independent variables. To control for potential confounders, a multiple logistic regression model with backward selection was used. Whose variables with level statistically significant P <0.25 on univariate analysis were entered jointly into a multivariate logistic regression. All statistical tests were two-tailed, and the significance level was declared at p<0.05 with 95% confidence level. Model fit was assessed using the Hosmer-Lemeshow goodness of fit test.

## Results

### Socio-demographic characteristics of respondents

A total of 194 respondents were interviewed with a response rate of 95%. One hundred and ten (56.7%), 55(28.9%) and 28(14.4%) of the respondents were from government, private, and community kindergarten respectively. Almost all study participants 193 (99.5%) were females. The mean age of the respondents was 29.3 with standard deviation of 6.5. The minimum and the maximum age of the respondents were 20 and 54 years respectively. Among respondents, 113 (58.2%) were married and 66 (34.0%) were singles while the others were divorced and widowed. Concerning educational level of the participants, about 64.9% of them were certificate holders, 22.7% of them hold diploma, and the remaining had bachelor degree. About half of the participants had less than five years work experience and one third of them had five to ten years’ experience while the others served for more than ten years. Of all the respondents, only one third of them had previous first aid training (Table 1).

**Table 1:** Socio-demographic characteristics of respondents.

Variables	No.	%
**Sex**		
Male	1	0.5
Female	193	99.5
**Age (in year)**		
20–24	54	27.8
25–29	56	28.9
30–34	45	23.2
35 and above	39	20.1
**Level of education**		
Certificate	126	64.9
Diploma	44	22.7
Degree	24	12.4
**Year of experience (in year)**		
Less than 5	96	49.5
5–10	69	35.6
Above 10	29	14.9
**Marital status**		
Married	113	58.2
Unmarried	66	34.0
Divorced	12	6.2
Widowed	3	1.5
**Type of kindergarten**		
Public	56	28.9
Private	110	56.7
Community	28	14.4
**Previous first aid training**		
Yes	62	32.0
No	132	68.0

### Knowledge of kindergarten teachers on first aid

All respondents were sentient about first aid. One hundred and three (53.1%), 43(22.2%) and 28 (14.4%) heard about first aid from health professional, media and health institution, respectively while the rest heard it from other sources like family and books. Only forty percent of the kindergarten teachers fall in knowledgeable range on knowledge questions that given in questionnaire. There was a variation in knowledge of first aid for different cases. Majority of the participants stated that nose bleeding is requires first aid care followed by choking and epilepsy. Only quarter of the respondents know the importance of first aid for breathing difficulty, fracture, and injury of neck and back (Table 2).

**Table 2:** Respondents’ response for cases requiring first aid.

Type of cases	No.	%
Nose bleeding	130	67.0
Choking	112	57.7
Epilepsy	108	55.7
Fainting	97	50.0
Swallowed poison	86	44.3
Burning	76	39.2
Human/animal bite	64	33.0
Breathing difficulty	54	27.8
Fracture	51	26.3
Neck and Back injury	41	21.1

Of all respondents, only 40.2% of them scored above mean for knowledge question. Knowledge of participant on specific cases was varied. For instance, the knowledge of the participants for epilepsy, choking, nosebleed, and back and neck injury was 35.6%, 37.6%, and 38.7%, respectively.

### Practice of kindergarten teachers on first aid

About eighty percent of the respondents encountered with children in need of first aid. Of these respondents, 89.7% of them had given first aid. One hundred and thirty (83.9%) participants faced child with epistaxis (nose bleed). Major of participants applied uninterrupted pressure by pressing nostrils together and about 78 (60.0%) of them allow the child to sit slightly forward and comfortably as first aid measures. For child with body bleeding, the main practice of respondents were pressing firmly with clean bandage to stop bleeding, bandaged bleeding wound without interfering circulation, and contacted responsible school authority and parent (Table 3).

**Table 3:** First aid practice of respondents on different cases.

Variables	No.	%
**Encountered children in need of first aid (n = 194)**		
Yes	155	79.9
No	39	20.1
**Given first aid (n = 155)**		
Yes	139	89.7
No	16	10.3
**Type of first aid required cases encountered (n = 155)**		
One type	46	29.7
Two types	51	32.9
Three and more types	58	37.4
**Children with difficulty of breathing (n = 23)**		
Called ambulance	4	17.4
Encouraged the child to sit quietly	21	91.3
Breathe slowly and deeply	6	26.1
Contacted responsible school authority and parent	16	69.6
**Children with fainting (n = 44)**		
Called EMS/Ambulance	5	11.4
Kept child on flat position	31	70.5
Loosen clothing around the neck and waist	24	54.5
Kept air way clear and monitored breathing	5	11.4
Gave nothing by mouth	25	56.8
Contacted responsible school authority and parent	28	63.6
**Nose bleeding/epistaxis (n = 130)**		
Called EMS/Ambulance	7	5.4
Placed child sitting comfortably with slightly forward	78	60.0
Laid on side with head raised on pillow	12	9.2
Applied uninterrupted pressure by pressing nostrils together	84	64.6
Applied ice to nose	24	18.5
Contacted responsible school authority and parent	33	25.4
**Bleeding on child’s body (n = 72)**		
Called EMS/Ambulance	4	5.6
Pressed firmly with clean bandage to stop bleeding	47	65.3
Elevated bleeding body part gently	12	16.7
Bandaged bleeding wound without interfering circulation	42	58.3
Covered child with blanket	6	8.7
Contacted responsible school authority and parent	33	45.8
**Children with seizure/epilepsy (n = 41)**		
Called EMS/Ambulance	4	9.8
Left the child for free movement	14	34.1
Moved surrounding objects to avoid injury	26	63.4
Avoided giving any drink/food by mouth	21	51.2
Kept air way clear by placing the child on the side	17	41.5
Contacted responsible school authority and parent	20	48.8
**Children with chocking (n = 44)**		
Called EMS/Ambulance	36	82.0
Checked for choking	18	43.9
Stood behind the child encircling the child’s chest by hands and squeezed	34	77.3
Continued until the object expelled	11	25.0
Contacted responsible school authority and parent	6	14.3
**Children with injured neck and back (n = 12)**		
Called EMS/ambulance	3	25.0
Checked child’s position immediately	9	75.0
Laid the child and restrict moving unless harm exacerbated if the child stayed there	3	25.0
Avoided head and neck movement and kept body straight	12	100.0
Contacted responsible school authority and parent	10	83.3

Among kindergarten teachers who faced children in need of first aid, about quarter of them had faced children with fainting. For child in faint the kindergartner practice was keeping the child on the flat position, contacting responsible body, giving nothing by mouth, and loosing clothing around the neck and waist with proportion 31 (70.5%), 28 (63.6%), 25 (56.8%), and 24 (54.5%) respectively. About 44 (28.4%) of respondents faced children with choking. Thirty-six (82.0%) stood behind the child encircling the child’s chest by hands and squeezed whereas 34 (77.3%) of them called ambulance. About 7.7% of respondents faced children with neck and back injury and almost all respondents avoided head and neck movement and kept body straight. In addition, for the neck and back injury majority of kindergartner contacted responsible body in kindergarten and parent, checked child’s position and called ambulance.

About fifteen percent of respondents faced children with difficulty of breathing. Majority of them encouraged the child to calm down and sit quietly as a first aid care. A few of them contacted responsible school authority and parent, ordered the child to breathe slowly and deeply and called for ambulance.

Forty-one (26.5%) of the respondents faced children with epilepsy. Kindergartners practice for this case was also varied. For instance, about 63.4% of the respondents had moved surrounding objects from the child 51.2% of them avoided giving any drink/food by mouth, and 34.1% of them, left the child for free movement as a first aid measure for epilepsy.

### Attitude of kindergarten teachers towards first aid

Majority of the respondents showed positive attitude towards giving and learning first aid. In the overall attitude assessment, three fourth of the respondent had positive attitude towards to first aid. About 88% of the respondents strongly agreed with the idea of giving first aid whereas few of the respondents were strongly disagreed with the idea of giving first aid is pleasant in kindergarten. Eighty-four percent of respondents strongly agreed as learning first aid is important for them. Almost all kindergarten teachers consider that giving first aid is responsibility of the teacher. About 11.3% of respondents disagree on idea of giving a special care for injured student in their academic works (Table 4).

**Table 4:** Respondents’ attitude towards first aid.

Variables	Strongly agree No. (%)	Agree No. (%)	Disagree No. (%)	Strongly Disagree No. (%)
Giving first aid at school is fair	171(88.2)	22(11.3)	1(0.5)	-
Giving first aid at school is pleasant	100 (51.6)	74(38.1)	7(3.6)	13(6.7)
Giving first aid is very good	154(79.4)	40(20.6)	-	-
It is good for me to learn first aid	165(85.1)	27(13.9)	2(1)	-
It is important for me to learn first aid	163(84.0)	28(14.4)	3(1.6)	-
It is responsibility of teacher to giving first aid care for children in need	171(88.1)	22(11.3)	1(0.6)	-
Giving a special care for injured children in academic work is appropriate	111(57.2)	61(31.4)	21(10.8)	1(0.6)

### Factors affecting knowledge of kindergarten teachers for first aid

Bivariate and multivariable logistic regression analyses were done to analyze factors associated with knowledge of participants for first aid after checking the fitness of the model for the variable by using Hosmer and Lemeshow test. In univariate logistic regression analysis, age of participants, year of experience, level of education, type of school where the participant serves, source of first aid information, attitude towards to first aid and previous first aid training were eligible for multivariate logistic regression. Those variables that were identified by univariate analysis were adjusted by backward stepwise logistic regression after including other variable according to the criteria discussed in method part to assess factors that independently affect first aid knowledge of participants.

In multivariable logistic regressions, age more than 35 years, year of experience, type of school, source of first aid information, and previous first aid training were found to have significant association with knowledge of first aid at 95% CI with P-value of <0.05. Participant older than 35 years had four times odds of being knowledgeable about first aid as compared to those who were 25 and younger age [AOR = 4.2, 95% CI: (1.02, 16.9)]. Kindergarten teachers who had five to ten years’ experience had about three times more knowledgeable than those teachers who had less than five years’ experience [AOR = 3.1, 95% CI: (1.2, 7.6)]. Similarly, participants with more than ten years’ experience had about four times more knowledgeable than those who have less than five years’ experience [AOR = 3.7, 95% CI: (1.06, 13.3)]. Teachers who were serving in private kindergarten had about sixty times more knowledgeable than teachers who serve in government kindergarten [AOR = 15.7, 95% CI: (5.3, 45.6)]. Teacher who had first aid training three times more knowledgeable than those without training [AOR = 3.1, 95% CI: (1.2, 7.7)]. In addition to this source of first aid information was also independently associated with knowledge of first aid. Participant who obtain first aid information from health professionals or health institute were about two times more likely knowledgeable than participants who got the information from other sources [AOR = 2.4, 95% CI: (1.1, 5.2)] (Table 5).

**Table 5:** Factors associated with knowledge of first aid among kindergarten teachers.

Variable	Knowledgeable participant (n = 78)	Not Knowledgeable participant(n = 116)	COR (95% CI)	AOR (95% CI)	P-Value
**Age (year)**					
25 and less	21	46	Ref.		
26–35	40	62	1.4 (0.7, 2.7)	0.97 (0.3, 2.7)	0.79
More than 35	17	8	4.6 (1.7, 12.5)	4.2 (1.02, 16.9)	0.046*
**Level of Education**					
Certificate	39	87	Ref.		
Diploma	22	22	2.2 (1.1, 4.5)		
Degree	17	7	5.4 (2.1, 14.1)		
**Year of experience**					
Less than 5	26	70	Ref.	Ref.	
5–10	35	34	2.8 (1.4, 5.3)	3.1 (1.2, 7.6)	0.015*
More than 10	17	12	3.8 (1.6, 9.1)	3.7 (1.06, 13.3)	0.040*
**Type of Kindergarten**					
Government	9	47	Ref.	Ref.	
Private	64	46	7.3 (3.2,16.3)	15.7 (5.3, 45.6)	<0.001*
Community	5	23	1.1 (0.3, 3.7)	3.24 (0.76, 13.7)	0.109
**Previous first aid training**					
Yes	34	28	2.4 (1.3, 4.5)	3.1 (1.2, 7.7)	0.018*
No	44	88	Ref.	Ref.	
**Attitude towards to first aid**					
Positive attitude	63	82	1.7 (0.9, 3.5)		
Negative attitude	15	34	Ref.		
**Source of information**					
Neither HP nor HI sources	26	53	Ref.	Ref.	
HP or HI sources	46	57	1.6 (0.9, 3.0)	2.4 (1.1, 5.2)	0.026*
HP or HI, and other source	6	6	2.0 (0.6, 6.9)	2.9 (0.6, 12.7)	0.15

HI: Health Institute; HP: Health professional;

*Shows significant association for multivariate logistic regressions at 95% CI;

Ref. = Shows reference

### Factors affecting kindergartners’ attitude towards to first aid

Kindergarten teacher attitude towards to first aid was assessed by using seven questions by using four point Likert scale. Logistic regression model was used to assess factors affecting kindergarten teachers’ attitude towards to first aid. Knowledge of first aid, year of experience, type of kindergarten the participants work, previous first aid training, source of information, and exposure of first aid requiring cases were screened by univariate binary logistic regression for multivariate logistic regression analysis. Variables independently associated with attitude of kindergarten teachers were year of experience, type of kindergarten where the participant work, previous first aid training, and exposure to first aid requiring cases.

Kindergarten teacher who had 5–10 years experience and more than 10 years had five times [AOR = 5.0, 95% CI: (2.1, 12.4)] and four times [AOR = 4.2, 95% CI: (1.3, 13.6)] more positive attitude for first aid than teachers who taught less than 5 years, respectively. Type of work place of participant also affected their attitude for first aid. Those teachers who taught in private kindergarten had about two times [AOR = 2.4, 95% CI: (1.1, 5.3)] more positive attitude than government kindergarten. Similarly, community kindergarten workers teacher had about four times [AOR = 3.7, 95% CI: (1.1, 12.0)] more positive attitude than those who taught in governmental kindergarten. Encountering first aid needing cases enhance attitude of kindergarten teachers by 2.6 folds [AOR = 2.6, 95% CI: (1.1, 5.9)] as compared to non exposed teachers. Previous first aid training was also another factors significantly associated with positive attitude (Table 6).

**Table 6:** Factors associated with attitude of first aid among kindergarten teachers.

Variables	Positive attitude (n = 145)	Negative attitude (n = 49)	COR (95% CI)	AOR (95% CI)	P-Value
**Knowledge of first aid**					
Knowledgeable	63	15	1.7 (0.9, 3.5)		
Not Knowledgeable	83	34	Ref.	Ref.	
**Year of experience**					
Less than 5	61	35	Ref.	Ref.	
5–10	61	8	4.37 (1.9, 10.2)	5.0 (2.1, 12.4)	<0.001*
More than 10	23	6	2.2 (0.8, 5.9)	4.2 (1.3, 13.6)	0.014*
**Type of Kindergarten**					
Government	37	19	Ref.	Ref.	
Private	86	24	1.8 (0.9, 3.8)	2.4 (1.1. 5.3)	0.038*
Community	22	6	1.9 (0.6, 5.4)	3.7 (1.1, 12.0)	0.032*
**Previous first aid training**					
Yes	41	21	0.5 (0.3, 1.0)	0.4 (0.2, 0.8)	0.014*
No	104	28	Ref.	Ref.	
**Source of information**					
Neither HP nor HI sources	63	16	Ref.		
HP or HI sources	73	30	0.6 (0.3, 1.2)		
HP or HI, and other source	9	3	0.8 (0.2, 3.1)		
**Exposure of children in need of first aid**					
Faced children in need of first aid	121	34	2.2 (1.1, 4.7)	2.6 (1.1, 5.9)	0.027*
Not faced children in need of first aid	24	15	Ref.	Ref.	

HI: Health Institute; HP: Health professional;

*Shows significant association for multivariate logistic regressions at 95% CI;

Ref. = Shows reference

## Discussion

In the present study, it was observed that all kindergarten teachers who participated in the study were heard about first aid. Majority of participants got the information from health professionals. However, when an inquiry was made in depth regarding first aid knowledge, about sixty percent of participants were found to be non knowledgeable. This implies that even though teachers are briefly introduced about first aid, they lack detailed knowledge about it. Our finding is in line with the study conducted in Egypt that the mean score of the respondents was found to be low [23]. It is also similar with the finding of a study done in U.S, Midwestern states that indicated most teachers have deficient knowledge of emergency care and basic life support modalities [18]. The finding of our study is also consistent with the finding of Abha City, Kingdom of Saudi Arabia [24] and Nigerian teachers’ knowledge towards epilepsy first aid [25].

According to the participants’ point of view, bleeding is the major case that needs first aid followed by epilepsy and choking while cases such as neck and back injury, breathing difficulty, and fracture were considered as less likely requires first aid care. The knowledge of participants for first aid was further assessed by using twelve questions related to specific cases that require first aid care. The cases are selected based on commonly observed injury in schools where children spend their major part of the day with their peers and teachers. For instant, only 35.6%, 42.3%, and 37.6%of participants know the main epilepsy, bleeding, and choking first aid management respectively. This finding is similar to the study done in China [21]. Children have high risk of injuries and emergencies cases due to their higher level of involvement in physical activities and other extra-curricular activities. Kindergarten teachers act as the guardians of these children and they need to be equipped with the adequate knowledge and practice regarding first aid. The level of knowledge in Port Said kindergarten teachers was low as compared to our finding [23]. This difference may be due to lack of effective training on pediatrics first aid.

In our study, participants’ main source information about first aid was health professional. In contrast, Zimbabwe studied finding showed that about half of the caregivers got first aid care information from family members followed by internet and books while few of them obtained information from mass media [26]. The observed difference may be due to difference in study populations’ profession, level of education, and exposure to training. In study conducted on evaluation of first aid knowledge on burns management amongst high risk groups, the source of information was mainly obtained from books and internet [27]. Similarly, Sönmezet al., (2014) reported as the most commonly source of first aid knowledge was the media [28]. In ours study, those kindergarten teacher obtain first aid information from health professional or health institute were about two times more knowledgeable than the others. This may be due to difference in strength obtained of information. Health professionals are mainly trained on the different aspect of health care systems and they are appropriated individual to transmit first aid information.

In the current study, only about one third of respondents had previous first aid training which is less than the study conducted in Midwestern USA, which show two third of respondents had previous first aid training [18]. Training accessibility of developing countries is less as compared to developed world. In our finding, training had positive association with knowledge of first aid. Kindergarten teachers who had first aid training were about three times more knowledgeable than teachers who had no first aid training. Observed low first aid knowledge in the present study may be due to less attention for kindergartner teachers training on first aid. Similarly, study in China revealed significant association between previous first aid training and knowledge [21]. On the other hand study conducted in Turkey showed no significant association between training and first aid knowledge [28]. The observed difference may be due to method and depth of training give for teachers.

This study also showed that older ages and long time experience were significantly associated with first aid knowledge of participants. Kindergarten teachers older than 35 years had four times more knowledge about first aid than those with age 25 years and less. Respondents who had service year between five to ten years were three times more likely knowledgeable than teachers who had less than five years work experience. Similarly, teachers who had more than 10 years work experience had about four times more knowledge than teachers with less than 5 years work experience. The more individuals stay in the service, the better accesses to the first aid training and the more exposure to cases which enhance desire of acquiring knowledge of first aid. Study conducted in Port Said also showed correlation between year of experience as kindergarten teacher and first aid knowledge [23].

The current study also showed that there is significant association between type of school where study participants taught and their knowledge for first aid. Those kindergarten teachers teaching in private kindergarten had about sixteen times more chance of being knowledgeable about first aid than those who teach in government kindergartens. This may be due to, that private school teachers in our study were more trained on first aid than government kindergarten teachers.

In this study majority of participant has positive attitude for first aid. Most respondents agreed that giving first aid was helpful; the vast majority believe the importance and usefulness of learning first aid. This result is in line with the study done in Shanghai, China, in which majority of the participants felt the importance of providing first aid and learning first aid [21]. In the overall attitude questions, about three fourth of the respondents had positive attitude against first aid. On the other hand, study conducted on attitude of first aid for epilepsy showed that largest proportion of respondents had negative attitudes before training [25]. Abulhamail et al (2014) also showed that teachers with good knowledge were less likely to have negative attitudes including and their attitude was highly correlated with their knowledge [29].

Factors affecting kindergarten teachers’ attitude towards to first aid were assessed. One of the factors was length of work experience. Those teachers who had more than five years work experience had more likely positive attitude than teachers who had less than five years work experience. The possible reason for this difference may be due to the acceptance and love of their work. When teachers stay in teaching for long time, they adaptation the environment and develop giving attention of their students. Private and community kindergarten teachers had less likely negative attitude for first aid as compared to governmental employees. This may be due to good access to first aid training in our study. In our study, teachers who face child in need of first aid have more likely positive attitude for first aid than none exposed teaches. Previous first aid training has significant association with positive attitude of participants. Similarly the other studies also showed that first aid training has positive association with attitude of teachers [25, 29].

About eighty percent of kindergarten teachers had exposure of first aid requiring cases. The number and type of first aid cases encountered by teaches varied. Slightly majority of teachers faced three and more different cases. As whole, proportion of kindergarten teachers who perform required activities for each case is very few. This indicates that majority of first aid care were not delivered for children. Likewise, study conducted in Sri Lanka, overall mean ±SD value that was scored for the practices section was 4.53±1.48 out of 10 scores. Only few individuals were able to obtain scores above mean in practice question [30]. Majority of practices were non scientific and traditionally transferred from family. For instance, in study conducted in Zimbabwe, caregivers’ major practice for burn was mainly cooling the burn injury with cold running water; some caregivers also applied eggs, margarine and traditional herbs [26].

This study has a number of limitations. One of the limitations is bias occurred as a result of study design (cross-sectional), because the study takes information at specified time-points and cause and effect association cannot be studied. Although different reducing mechanisms were used, social desirability bias is the other potential limitations of this study. Recall bias and selection bias due to small sample size can be observed. Other limitation was absence of uniformity and variation in type of first aid requiring cases, results difficulty in assessment of factors associated with practice of participant. Since our study was conducted in limited study area, representativeness and generalizability of the study result may be compromised. Finally, lack of sufficient similar study made limit comparison with other study. To reduce these potential bias different mechanisms were used. Some of these are performing pre-test before actual data collection. To obtain genuine response, the aim of the study was discussed with respondents, and to reduce recall bias, the recall period was reduced to previous 12 months.

## Conclusion

The knowledge of kindergarten teachers towards first aid is found to be low in spite of almost of the respondents had positive attitude towards first aid. Majority of the respondents exposed to children in need of first aid and practice of kindergartners was mostly not in line standards. Having more than five years services, teachers with older age, having previous first aid training, obtaining fist aid information from health professional or health institutes, and working in private kindergarten were significantly associated with first aid knowledge. Similarly, attitude of kindergartners were associated with more than five years experience, type of kindergarten, previous first aid training, and exposure first aid requiring cases. It would be better if schools have a link with health institutions for first aid training and immediate referral in cases of student injury. It would also be better if the ministry of education include first aid course in the kindergarten teachers’ education.

## Supporting information

S1 File Questionnaire. (DOCX)
