# Supplementary material for: First aid knowledge, attitude, practice, and associated factors among kindergarten teachers of Lideta sub-city Addis Ababa, Ethiopia
Source: PLoS One. 2018 Mar 13;13(3):e0194263. doi: 10.1371/journal.pone.0194263 (PMC5849320; doi:10.1371/journal.pone.0194263)
Supplement: S1 File — (DOCX) [file pone.0194263.s001.docx]

# S1 File. Questionnaire

**Participant information sheet**

Title of the research project: “First aid knowledge, attitude, practice, and associated factors among kindergarten teachers of Lideta sub-city Addis Ababa, Ethiopia”.

First of all we would like to thank you in advance for your cooperation and consent in participation in this study. Please read about the general information of the study. If you have any question regarding the study please ask freely.

The results of the study will be used as base line information to design appropriate intervention strategies to increase kindergarten teachers’ knowledge, attitude and practice of first aid. The questionnaire contains both closed and open ended questions and will be provided in self-administered form. You are therefore kindly requested to provide genuine answers to the questions. The information you provide is confidential and is used only for the purpose of this study. If you have any question, don‘t hesitate to ask the data collector. Your cooperation and participation until the completion of the questionnaire is very necessary for the successful completion of the study.

We therefore ask your genuine willingness. However, you have the right to refuse if you are not voluntary to participate by making thick mark in ‘No’ in the box below.

Are you voluntary Yes No

Thank you in advance for your cooperation

Data collector ____________________, date ____________sign: __________

Questionnaire code: _________________

**Persons to contact:**

If you have any question to ask, please contact

Gemechu Ganfure

Tel: +251-929381738/+251-942108039, Email = [gemesda7@gmail.com](mailto:gemesda7@gmail.com)

## English Version Questionnaires

**Part I: Socio-demographic characteristics of kindergarten teachers in Lideta Sub City, Addis Ababa**

| **Ser. no** | **Socio-Demographic** | **Response** | **Remark** |
| --- | --- | --- | --- |
| **1** | Sex | 1. Male 2. Female |  |
| **2** | Age | _______in years |  |
| **3** | Level of education | 1. Certificate 2. Diploma 3. Degree 4. Masters 5. Other (specify) ____ |  |
| **4** | Marital status | 1. Married 2. Single 3. Divorced 4. Widowed/ |  |
| 5 | Service year | 1. <5 yrs. 2. 5-10 yrs. 3. >10 yrs. |  |
| **6** | Type of kindergarten | 1. Government 2. Private 3. Public 4. Others |  |
| **7** | Do you have training on first aid? | 1. Yes 2. No |  |

**Part II: Questions to assess kindergarten teachers first aid knowledge in Lideta Sub City, Addis Ababa**

1. Have you ever heard about first aid?
2. Yes
3. No
4. If yes for Q 1, from where did u hear? **You can choose more than one option.**
5. Family
6. books
7. media
8. health professionals
9. health institution
10. others, specify___________________
11. Yes for Q 1, what is first aid?
12. The immediate care given for a person who sustained any injury or accident before the victim arrive health institution.
13. The care given only in health institution
14. The care given only by health profession
15. Other specify, ________________________________________
16. What type of injuries/accidents need first aid? You can choose more than one optionከዚህ
17. Bleeding
18. Fracture
19. Epilepsy
20. Human/animal bite
21. Burning
22. Nose bleeding
23. Choking
24. Neck and back injury
25. Fainting
26. Swallowed poison
27. Breathing difficulty
28. Others, specify……………………
29. One measure to stop bleeding is pressing firmly with clean bandage on the bleeding part?
    1. True
    2. False
    3. Not know
30. Giving nothing by mouth is one of the first aid measures for fainting child?
31. True
32. False
33. Not know
34. One of the first aid measures for epileptic child is keeping air way clear by placing the child on the side?
    1. True
    2. False
    3. Not know
35. Standing behind the child encircling the child’s chest by hands and squeezing is the first aid measure for choking child?
    1. True
    2. False
    3. Not know
36. For child with neck and back injury avoiding head and neck movement and keeping body straight is one measure of first aid.
    1. True
    2. False
    3. Not know
37. In case child has bitten by his friend, cleansing wound with soap and water for 5 minutes is one measure of first aid for human bite.
    1. True
    2. False
    3. Not know
38. One of the first aid measures for nose bleed/epistaxis is placing student sitting comfortably with slightly forward and applying uninterrupted pressure by pressing nostrils together.
    1. True
    2. False
    3. Not know
39. Encouraging the child to sit quietly, breath slowly and deeply in through the nose and out through the mouth is first aid measure for the child with difficulty of breathing?
    1. True
    2. False
    3. Not know

**Part III: questions to assess attitude of kindergarten teachers towards first aid teachers in Lideta Sub City, Addis Ababa**

| 1. Giving first aid at school is fair | 1. Strongly agree 2. Agree 3. Disagree 4. Strongly disagree |
| --- | --- |
| 2.Giving first aid at school is pleasant | - - 1. Strongly agree     2. Agree     3. Disagree     4. Strongly disagree |
| 1. Giving first aid is not good | 1. Strongly agree 2. Agree 3. Disagree 4. Strongly disagree |
| 1. It is good for me to learn first aid | 1. Strongly agree 2. Agree 3. Disagree 4. Strongly disagree |
| 1. It is important for me to learn first aid | 1. Strongly agree 2. Agree 3. Disagree 4. Strongly disagree |
| 1. It is responsibility of teacher to giving first aid care for children in need | 1. Strongly agree 2. Agree 3. Disagree 4. Strongly disagree |
| 1. Giving a special care for injured children in academic work is appropriate | 1. Strongly agree 2. Agree 3. Disagree 4. Strongly disagree |

**Part IV: Questions to assess practice of kindergarten teachers on first aid in Lideta Sub City, Addis Ababa**

| - - - 1. Have you ever faced a child with in need of first aid in your school? |  | a. yes  b. no |
| --- | --- | --- |
| - - - 1. If yes for Q 1, did you give first? |  | - - - - 1. Ye         2. No |
| - - - 1. If yes for Q 2, what was your first action? | - - - - 1. Called 939 (Country emergency call)         2. Transferred to hospital         3. Gave first aid         4. Transferred to police station         5. Other, specify, __________ |  |
| - - - 1. Have you ever faced a child with difficulty of breathing? |  | a. Yes  b. No  If NO skip to Q. 6 |
| - - - 1. If your answer **for Q 4 is “yes”**, what did you do?   **You can choose more than one option** | 1. Called EMS/ambulance | a. Yes  b. No |
|  | 1. Encouraged the student to sit quietly, | a. Yes  b. No |
|  | 1. Breath slowly and deeply in through the nose and out through the mouth | a. Yes  b. No |
|  | 1. Contacted responsible school authority and parent or legal guardian | a. Yes  b. No |
|  | 1. Others, specify _____________________ |  |
| - - - 1. Have you ever faced a child with fainting? |  | 1. Yes 2. No   If NO skip to Q. 8 |
| - - - 1. If your answer **for Q 6 is “yes”**, what did you do?   **You can choose more than one option** | 1. Called EMS/Ambulance | a. Yes  b. No |
|  | 1. Kept student on flat position | a. Yes  b. No |
|  | 1. Loosen clothing around the neck and waist | a. Yes  b. No |
|  | 1. Kept air way clear and monitored breathing | a. Yes  b. No |
|  | 1. Gave nothing by mouth | a. Yes  b. No |
|  | 1. Contacted responsible school authority and parent or legal guardian | a. Yes  b. No |
|  | 1. Others, specify |  |
| - - - 1. Have you ever faced a child with bleeding from his/her nose? |  | a. Yes  b. no  If NO skip to Q. 10 |
| - - - 1. If your answer **for Q 8 is “yes”**, , what did you do? **You can choose more than one option** | - 1. Called EMS/ambulance | a. Yes  b. No |
|  | - 1. Placed student sitting comfortably with slightly forward | a. Yes  b. No |
|  | - 1. Laid on side with head raised on pillow | a. Yes  b. No |
|  | - 1. Applied uninterrupted pressure by pressing nostrils together | a. Yes  b. No |
|  | - 1. Applied ice to nose | a. Yes  b. No |
|  | - 1. Contacted responsible school authority and parent or legal guardian | a. Yes  b. No |
|  | - 1. Others, ___________ | a. Yes  b. No |
| - - - 1. Have you ever faced a child with bleeding on his/her body? |  | a. Yes  b. No  If NO skip to Q. 12 |
| - - - 1. If your answer **for Q 10 is “yes”**, what did you do?   **You can choose more than one option** | 1. Called EMS/Ambulance | a. Yes  b. No |
|  | 1. Pressed firmly with clean bandage to stop bleeding | a. Yes  b. No |
|  | 1. Elevated bleeding body part gently | a. Yes  b. No |
|  | 1. Bandaged bleeding wound without interfering circulation | a. Yes  b. No |
|  | 1. Covered student with blanket | a. Yes  b. No |
|  | 1. Contacted responsible school authority and parent or legal guardian | a. Yes  b. No |
|  | 1. Others, __________ |  |
| - - - 1. Have you ever faced a child with seizure/epilepsy? |  | a. Yes  b. No  If NO skip to Q. 14 |
| - - - 1. If your answer **for Q 12 is “yes”**, ,what did you do?   **You can choose more than one option** | 1. Called EMS/Ambulance | a. Yes  b. No |
|  | 1. Placed on the floor | a. Yes  b. No |
|  | 1. Left the child for free movement | a. Yes  b. No |
|  | 1. Moved surrounding objects to avoid injury | a. Yes  b. No |
|  | 1. Avoided giving any drink/food by mouth | a. Yes  b. No |
|  | 1. Kept air way clear by placing the child on the side | a. Yes  b. No |
|  | 1. Contacted responsible school authority and parent or legal guardian | a. Yes  b. No |
|  | 1. Others, _______ |  |
| - - - 1. Have you ever faced a child with chocking: |  | a. Yes  b. No  If NO skip to Q. 10 |
| - - - 1. If yes, what did you do?   **You can choose more than one option** | - 1. Called EMS/Ambulance | a. Yes  b. No |
|  | - 1. Checked for choking | a. Yes  b. No |
|  | - 1. Stood behind the child encircling the child’s chest by hands and squeezed | a. Yes  b. No |
|  | - 1. Continued until the object expelled | a. Yes  b. No |
|  | - 1. Contacted responsible school authority and parent or legal guardian | a. Yes  b. No |
|  | 1. Others, ___________ |  |
| - - - 1. Have you ever faced a child with injured neck and back? |  | a. Yes  b. No |
| - - - 1. If your answer **for Q 16 is “yes”**, what did you do?   **You can choose more than one option** | 1. Called EMS/ambulance | a. Yes  b. No |
|  | 1. Checked student’s position immediately | a. Yes  b. No |
|  | 1. Laid the student and restrict moving unless harm exacerbated if the students stayed there | a. Yes  b. No |
|  | 1. Avoided head and neck movement and kept body straight | a. Yes  b. No |
|  | 1. Contacted responsible school authority and parent or legal guardian | a. Yes  b. No |
|  | 1. Others, ___________ | a. Yes  b. No |

**Thank you for your cooperation!!!!**

## Amharic version questionnaire

**ክፍል1: በ አዲስ አባባ፡ ልደታ ክ/ከተማ፤ የዐፀደ ህፃናጽ መምኅራን ማህበረሰባዊና ግላዊ ነክ ኁኔታዎችን የሚመለከቱ ጥያቄዎች**

| ተራ ቁጥር | ማህበረሰባዊና ግላዊ | **መላስ** | **ማርመራ** |
| --- | --- | --- | --- |
| **1** | ፆታ | 1. ወንድ 2. ሴት |  |
| **2** | ዕድሜ | ___በ ዓመት |  |
| **5** | የትምህርት ደረጃ | 1. ሰርትፍከየት 2. ዲፕሎማ 3. ዲግሪ 4. ማስተርስ 5. ሌላ ካለይ ጥቀሱ____ |  |
| **6** | የጋብቻ ሁኔታ | 1. ያገባች/ያገባ 2. ያላገባ/ያላገባች 3. /የፈታ/የፈታች 4. / የሞተባት |  |
| 8 | ያገልግሎት ዘመን | 1. <5 .ዓመት 2. 5-10 .ዓመት 3. 10-15 .ዓመት 4. >15ዓመት |  |
| **9** | የት/ት ቤቱ ዓይነት | 1. የመንግስት 2. የግል 3. የህዝብ 4. ሌላ |  |
| **10** | ከዚህ በፊት የመጀመሪያ ህክምና ዕርዳታ ስልጠና ወስደዉ ያዉቃ? | 1. አዎ 2. አይ |  |

**ክፍል 2፡ በ አዲስ አባባ፡ ልደታ ክ/ከተማ፤ የዐፀደ ህፃናጽ መምኅራን ስለ መጀመሪያ ህክምና ዕርዳታ የግንዛቤ ጥያቄዎች**

1. ከዚህ በፊት ስለ መጀመሪያ ህክምና ዕርዳታ ሰምተዉ ያዉቃሉ?
   1. አዎ
   2. አይ
2. ለጥያቄ ቁጥር 1 ምላሽዎ “አዎ” ከሆነ ስለ መጀመሪያ ህክምና ዕርዳታ የሰሙት ከየት ነዉ?
3. ከቤተሰብ
4. ከመጽሃፍት
5. ከመገናኛ ብዙሃን
6. ከጤና ባለሙያ
7. ከጤና ተቋም
8. ሌላ ካለ ይጥቀሱ ___________________
9. ለጥያቄ ቁጥር 1 ምላሽዎ “አዎ” ከሆነ የመጀመሪያ ህክምና ዕርዳታ ሲባል ምን ይመስልዎታል?
   1. ድነገተኛ አደጋ የደረሰበት ሰዉ ጤና ተቋም ከመድረሱ በፊት ወይም ወድያዉኑ የሚደረግለት የጤና ዕርዳታ
   2. በጤና ተቋም ዉስጥ ብቻ የሚሰጥ ዕርዳታ
   3. በጤና ባለሙያ ዉስጥ ብቻ የሚሰጥ ዕርዳታ
   4. ሌላ ካለይጥቀሱ ___________________
10. ከዚህ በታች ከተዘረዘሩት አደጋዎች ዉስጥ የትኞቹ የመጀመሪያ ሕክምና ዕርዳታ ያስፈልጋቸዋል ብለዉ ያስባሉ? **(ከአንድ በላይ ምላሽ መስጠ ይችላሉ)**
11. መድማት
12. ስብራት
13. የሚጥል በሽታ
14. በሰዉ/በእንስሳት መነከስ
15. ቃጠሎ
16. ነስር
17. ትንታ
18. የአንገትና የጀርባ ጉዳት
19. ራስን መሳት
20. መርዘማ ነገሮችን መዉሰድ
21. የመተንፈስ ችግር
22. ሌላ ካለ ይጥቀሱ_____________
23. መድማትን ለማቆም ከምንወስዳቸዉ የመጀመሪያ ሕክምና ዕርዳታ ዕርምጃዎች ዉስጥ አነዱ በመድማት ላይ ያለዉን ቦታ በንጹህ ጨርቅ (bandage) አጥብቆ መያዝ መሆኑን ያዉቃሉ?
    1. እዉነት
    2. ዉሸት
    3. አላዉቅም
24. ራሱን ስቶ ላለ ልጅ በኣፍ ምንም አይነት ፈሳሽ ወይም ምግብ እንዳይወስድ ማድረግ በመጀመሪያ ሕክምና ዕርዳታ ከሚወሰዱ ዕርምጃዎች አንዱ መሆኑን ያዉቃሉ?
    1. እዉነት
    2. ዉሸት
    3. አላዉቅም
25. በሚጥል በሽታ መክንያት ለወደቀ ልጅ ከምንወስዳቸዉ የመጀመሪያ ሕክምና ዕርዳታ ዕርምጃዎች አንዱ ልጁን በጎኑ በማስተኛት የአየር በበቧንቧዉ ክፈት ሆኖ እንዲቆይ ማድረግ መሆኑን ያዉቃሉ?
    1. 1. እዉነት
    2. ዉሸት
    3. አላዉቅም
26. የትንታ አደጋ ላጋጠመዉ ልጅ ከልጁ ጀርባ በመሆን ሁለት እጅን በማጣመር የልጁን ደረት በጥንቃቄ በመጫን የገባዉ ባዕድነገር እንዲወጣ መሞከር በመጀመሪያ ሕክምና ዕርዳታ ከሚወሰዱ ዕርምጃዎች አንዱ መሆኑን ያዉቃሉ?
    1. እዉነት
    2. ዉሸት
    3. አላዉቅም
27. የአንገትና የጀርባ ድንገተኛ አደጋ ላጋጠመዉ ልጅ ከምንወስዳቸዉ የመጀመሪያ ሕክምና ዕርዳታ ዕርምጃዎች አንዱ የልተገባ እንቅስቃሴ ከማድረግ መቆጠብና የልጁ ሰዉነት ቀጥ ብሎ እነዲቆይ ማድረግ መሆኑን ያዉቃሉ?
    1. እዉነት
    2. ዉሸት
    3. አላዉቅም
28. አንድ ልጅ በጓደኛዉ ቢነከስ የተነከሰዉን ቦታ በዉሃና ሳሙና ለአምስት ደቂቃ መጠብ በሰዉ ለመነከስ ከሚደረጉ የመጀመሪያ ሕክምና ዕርዳታ አንዱ መሆኑን ያዉቃሉ?
    1. እዉነት
    2. ዉሸት
    3. አላዉቅም
29. ነስር (የአፍንጫ መድማት) ላገጠመዉ ልጅ ከሚደረጉ የመጀመሪያ ሕክምና ዕርዳታዎች ዉስጥ ልጁን አመቻችቶ በተወሰነ መልኩ ወደፊት ጋደል አርጎ ማስቀመጥና አፍነጫዉን ተጭኖ መያዝ መሆኑን ያዉቃሉ?
    1. እዉነት
    2. ዉሸት
    3. አላዉቅም
30. የመተንፈስ ችግር ላጋጠመዉ ልጅ ከሚደረጉ የመጀመሪያ ሕክምና ዕርዳታዎች ዉስጥ ልጁ ተረጋግቶ እዲቀመጥ ማድረግ፣ ቀስ አድረጎ በአፍንጫዉ በደንብ አየር እንድያ ስገባና በአፍ እንዲያስወጣ ማድረግ እንደሆኑ ከዚህ በፊት ያዉቃሉ?
    1. እዉነት
    2. ዉሸት
    3. አላዉቅም

**ክፍል 3፡ በአዲስ አባባ፡ልደታ ክ/ከተማ፤ በዐፀደ ህፃናት የሚገኙ መምህን የመጀመሪያ ህክምና ዕርዳታ አመለካከት የሚመለከቱ ጥያቄዎች**

| 1. ሙኃለ-እፃናጽት ዉስጥ የመጀመሪያ ሕክምና ዕርዳታ ማድረግ ጥሩ ነዉ፡፡ | 1. በጣም እስማማለሁ 2. እስማማለሁ 3. አልስማማም 4. በጣም አልስማማም |
| --- | --- |
| 1. ሙኃለ-እፃናጽት ዉስጥ የመጀመሪያ ሕክምና ዕርዳታ ማድረግ አመች ይደለም | 1. በጣም እስማማለሁ  2.እስማማለሁ  3.አልስማማም  4.በጣም አልስማማም |
| 1. የመጀመሪያ ሕክምና ዕርዳታ ማድረግ ጥሩ አይደለም፡፡ | 1. በጣም እስማማለሁ 2. እስማማለሁ 3. አልስማማም 4. በጣም አልስማማም |
| 1. የመጀመሪያ ሕክምና ዕርዳታ ማድረግ በጣም ጥሩ ነዉ፡፡ | 1. በጣም እስማማለሁ 2. እስማማለሁ 3. አልስማማም 4. በጣም አልስማማም |
| 1. የመጀመሪያ ሕክምና ዕርዳታ መማር ጥሩ ነዉ፡፡ | 1. በጣም እስማማለሁ 2. እስማማለሁ 3. አልስማማም 4. በጣም አልስማማም |
| 1. የመጀመሪያ ሕክምና ዕርዳታ መስጠጥ የመምህር ግደታነዉ፡፡ | 1. በጣም እስማማለሁ 2. እስማማለሁ 3. አልስማማም 4. በጣም አልስማማም |
| 1. ለተጎዱ እፃናጽት የተየጥንቃቀ ማደረግ ተገቢ ነዉ፡፡ | 1. በጣም እስማማለሁ 2. እስማማለሁ 3. አልስማማም 4. በጣም አልስማማም |

**ክፍል 4፡ በአዲስ አባባ፡ልደታ ክ/ከተማ፤ በዐፀደ ህፃናት የሚገኙ መምህን የመጀመሪያ ህክምና ዕርዳታ ተግባርን የሚመለከቱ ጥያቄዎች**

| - 1. በሚሰሩበት በዐፀደ ህፃናት ት/ት ቤት ዉስጥ የመጀመሪያ ህክምና ዕረዳታ የሚያስፈልገዉ ልጅ ዐጋጥሞት ያዉቃሉን? |  | 1. አዎ  2. አይ |
| --- | --- | --- |
| 1. ለጥያቄ ቁ.1 ምላሽዎ “አዎ” ከሆኔ የመጀመሪያ ህክምና ዕርዳታሰጥተዋል? |  | 1. አዎ  2. አይ |
| 1. ለጥያቄ ቁ.2 ምላሽዎ “አዎ” ከሆኔ መጀመሪያ የወሰዱት ዕረምጃ ምንድር ነበር? | - - - - 1. አምቡላንስ ጠረሁ/ደወልኩ         2. ወደ ጤና ተቋም እንዲሄድ አደረኩ/ወሰድኩ         3. የመጀመሪያ ህክምና ዕርዳታ ሰጠሁ         4. ወደ ፖሊስ ጣቢያ እንዲሄድ አደረኩ         5. , ለላ ካለ የጥቀሱ __________ |  |
| 1. ደንገተኛ የመተንፈስ ችግር ያጋጠመዉ ልጅ አጋጥሞት ያዉቃሉን? |  | 1. አዎ  2. አይ  ምላሽዎ **“አይ”** ከሆኔ ወደ ጥያቄ ቁ.6 ይሂዱ |
| 1. ለጥያቄ ቁ.4 ምላሽዎ “አዎ” ከሆኔ ያደረጉት ነገር ምን ነበር? **ከአንድ ምላሽ በለይ መስጠት ይችላሉ** | - 1. አመቡላንስ ጠረሁ | 1. አዎ  2. አይ |
|  | - 1. ልጁ ተረጋግቶ እንዲቀመጥ አደረኩ | 1. አዎ  2. አይ |
|  | - 1. በዝግታ ብዙ አየር በአፍንጫዉ እያስገባ በአፉ እንዲያወጣ አደረኩ | 1. አዎ  2. አይ |
|  | - 1. የትምህርት ቤቱ ሃላፊና የልጁ ወላጅ/አሳዳጊ እንዲያዉቁ አደረኩ | 1. አዎ  2. አይ |
|  | - 1. ሌላ ካለ ይጥቀሱ---------------- |  |
| 1. ራሱን በድንገት የሳተ ልጅ አጋጥሞት ያዉቃሉን? |  | 1. አዎ  2. አይ  ምላሽዎ **“አይ”** ከሆኔ ወደ ጥያቄ ቁ.8 ይሂዱ |
| 1. ለጥያቄ ቁ.6 ምላሽዎ “አዎ” ከሆኔ ያደረጉት ነገር ምን ነበር? **ከአንድ ምላሽ በለይ መስጠት ይችላሉ** | - 1. አመቡላንስ ጠረሁ | 1. አዎ  2. አይ |
|  | - 1. ልጁን የተስተካከለ ቦታ ላይ እዲተኛ አደረኩ | 1. አዎ  2. አይ |
|  | - 1. አንገቱና ወገቡ አካባቢ ያሉ አልባሳትን እንዲላሉ አደረኩ | 1. አዎ  2. አይ |
|  | - 1. የአየር ቧንቧዉ ክፍት እነዲሆን በማድረግ አተነፋፈሱን ተከታተልኩ | 1. አዎ  2. አይ |
|  | - 1. በአፉ ምንም አይነት ፈሳሽና ምግብ እንዳይወስድ አደረኩ | 1. አዎ  2. አይ |
|  | - 1. የትምህርት ቤቱ ሃላፊና የልጁ ወላጅ/አሳዳጊ እንዲያዉቁ አደረኩ | 1. አዎ  2. አይ |
|  | - 1. ሌላ ካለ ይጥቀሱ___________________ |  |
| 1. በአፍንጫዉ ደም አየፈሰሰዉ (እያነሰረዉ) ያለ ልጅ አጋጥምዎት የዉቃሉን |  | 1. አዎ  2. አይ  ምላሽዎ **“አይ”** ከሆኔ ወደ ጥያቄ ቁ.10 ይሂዱ |
| 1. ለጥያቄ ቁ.8 ምላሽዎ “አዎ” ከሆኔ ያደረጉት ነገር ምን ነበር? **ከአንድ ምላሽ በለይ መስጠት ይችላሉ** | - 1. አመቡላንስ ጠረሁ | 1. አዎ  2. አይ |
|  | - 1. ልጁን አመቻች በማስቀመጥ በትንሹ ወደፊት ዘንበል (ጋደል) እንዲል አደረኩ | 1. አዎ  2. አይ |
|  | - 1. በጎኑ እነዲተኛ በማድረግ ጭንቅላቱን በትራስ ደገፍኩት | 1. አዎ  2. አይ |
|  | - 1. አፍንጫዉነ ተጭኘ በመያዝ እነዳይደማ አደረኩት | 1. አዎ  2. አይ |
|  | - 1. በረዶ አፍንጫዉ ላይ ደረኩ | 1. አዎ  2. አይ |
|  | - 1. የትምህርት ቤቱ ሃላፊና የልጁ ወላጅ/አሳዳጊ እንዲያዉቁ አደረኩ | 1. አዎ  2. አይ |
|  | - 1. ሌላ ካለ ይጥቀሱ___________ | 1. አዎ  2. አይ |
| 1. በድንገተኛ ጉዳት ከሰዉነቱ እየደማ ያለ ልጅ አጋጥሞት ያዉቃሉን? |  | 1. አዎ  2. አይ  ምላሽዎ **“አይ”** ከሆኔ ወደ ጥያቄ ቁ.12 ይሂዱ |
| 1. ለጥያቄ ቁ.10 ምላሽዎ “አዎ” ከሆኔ ያደረጉት ነገር ምን ነበር? **ከአንድ ምላሽ በለይ መስጠት ይችላሉ** | - 1. አመቡላንስ ጠረሁ | 1. አዎ  2. አይ |
|  | - 1. በመድማት ላይ ያለዉን ቦታ በንፁህ ጨርቅ (bandage) በመጫን እዳይደማ አደረኩ | 1. አዎ  2. አይ |
|  | - 1. በመድማት ላይ ያለዉነ አካል ከፍ እንዲል አደረኩ | 1. አዎ  2. አይ |
|  | - 1. በንፁህ ጨርቅ (bandage) የሰዉነቱን የደም ፍሰት በማያስተጓጉል መልኩ አሰርኩት(ሸፈንኩት) | 1. አዎ  2. አይ |
|  | - 1. ልጁ ብርድ ልብስ እንዲለብስ አደረኩት | 1. አዎ  2. አይ |
|  | - 1. የትምህርት ቤቱ ሃላፊና የልጁ ወላጅ/አሳዳጊ እንዲያዉቁ አደረኩ | 1. አይ 2. አዎ |
|  | - 1. ሌላ ካለ ይጥቀሱ____________ |  |
| 1. በሚጥል በሽታ ወድቆ እያነቀጠቀጠዉ ያለ ልጀ አጋጥሞት ያዉቃሉን? |  | 1. አዎ  2. አይ  ምላሽዎ **“አይ”** ከሆኔ ወደ ጥያቄ ቁ.14 ይሂዱ |
| 1. ለጥያቄ ቁ.12 ምላሽዎ “አዎ” ከሆኔ ያደረጉት ነገር ምን ነበር? **ከአንድ ምላሽ በለይ መስጠት ይችላሉ** | 1. አመቡላንስ ጠረሁ | 1. አዎ  2. አይ |
|  | 1. ወለሉ ላይ እንዲተኛ አደረኩ | 1. አዎ  2. አይ |
|  | 1. የሚያንቀጠቅጠዉ እስኪተዉ ልጁን ነፃ ሆኖ እዲንቀሳቀስ አደረኩ | 1. አዎ  2. አይ |
|  | 1. በዙሪያዉ ያሉ ጉዳት ሊያደርሱ የሚችሉ ዕቃዎችን አስወገድኩ | 1. አዎ  2. አይ |
|  | 1. በአፍ ምንም አይነት ፈሳሽም ሆነ ምግብ እንዳይወስድ አደረኩ | 1. አዎ  2. አይ |
|  | 1. ልጁን በጎኑ በማስተኛት የአየር ቧንቧዉ ክፍት ሆኖ እንዲቆይ አደረኩ | 1. አዎ  2. አይ |
|  | 1. የትምህርት ቤቱ ሃላፊና የልጁ ወላጅ/አሳዳጊ እንዲያዉቁ አደረኩ | 1. አዎ  2. አይ |
|  | 1. ሌላ ካለ ይጥቀሱ_______________ |  |
| 1. ትንታ ያጋጠመዉ ልጅ አግኝተዉ ያዉቃሉን |  | 1. አዎ  2. አይ  ምላሽዎ **“አይ”** ከሆኔ ወደ ጥያቄ ቁ.16 ይሂዱ |
| 1. ለጥያቄ ቁ.14 ምላሽዎ “አዎ” ከሆኔ ያደረጉት ነገር ምን ነበር? **ከአንድ ምላሽ በለይ መስጠት ይችላሉ** | - 1. አመቡላንስ ጠረሁ | 1. አዎ  2. አይ |
|  | - 1. ከልጁ ጀርባ በመሆን ሁለት እጅን በማጣመር የልጁን ደረት በጥንቃቄ በመጫን የገባዉ ባዕድነገር እንዲወጣ ሞከርኩ | 1. አዎ  2. አይ |
|  | - 1. የገባዉ ነገር አስኪወጣ መጫኑን ቀጠልኩ | 1. አዎ  2. አይ |
|  | - 1. የትምህርት ቤቱ ሃላፊና የልጁ ወላጅ/አሳዳጊ እንዲያዉቁ አደረኩ | 1. አዎ  2. አይ |
|  | 1. ሌላ ካለ ይጥቀሱ___________ |  |
| 1. ድንገት የአንገትና የጀርባ ጉዳት ያጋጠመዉ ልጅ አጋጥሞት ያዉቃሉን? |  | 1. አዎ  2. አይ |
| 1. ለጥያቄ ቁ.16 ምላሽዎ **“አዎ”** ከሆኔ ያደረጉት ነገር ምን ነበር? **ከአንድ ምላሽ በለይ መስጠት ይችላሉ** | 1. አመቡላንስ ጠረሁ | 1. አዎ  2. አይ |
|  | 1. የልጁን አወዳደቅ (አኳሃን) በፍጥነት ለማገናዘብ ሞከርኩ | 1. አዎ  2. አይ |
|  | 1. ልጁን አረጋግቶ በማሳረፍ ጉዳቱን ሊያባብሱ የሚችሉ አላስፈላጊ እነቅስቃሴዎችን አስወገድኩ | 1. አዎ  2. አይ |
|  | 1. አንገቱና ወገቡ እንዳይንቀሳቀስ በማድረግ ሰዉነቱ ቀጥ ባለ ሁኔታ እንዲቆይ አደረኩ | 1. አዎ  2. አይ |
|  | 1. የትምህርት ቤቱ ሃላፊና የልጁ ወላጅ/አሳዳጊ እንዲያዉቁ አደረኩ | 1. አዎ  2. አይ |
|  | 1. ሌላ ካለ ይጥቀሱ___________ | 1. አዎ  2. አይ |
